# The RAP study, report 4: morphological and topographical characteristics of multifocal macular neovascularization type 3

**DOI:** 10.1007/s00417-021-05332-8

**Published:** 2021-08-26

**Authors:** Bilal Haj Najeeb, Gabor G. Deak, Stefan Sacu, Ursula Schmidt-Erfurth, Bianca S. Gerendas

**Affiliations:** Vienna Reading Center, Department of Ophthalmology, Medical University of Vienna, Waehringer Guertel 18-20, 1090 Vienna, Austria

**Keywords:** Retinal angiomatous proliferation, Macular neovascularization type 3, Neovascular age-related macular degeneration, Fluorescein angiography, Optical coherence tomography

## Abstract

**Purpose:**

To report on the morphological characteristics and regional distribution of multifocal macular neovascularization type 3 (mMNV3).

**Methods:**

Twenty-two consecutive eyes of 21 patients with mMNV3 were included using multimodal imaging. The count and stage of lesions of all MNV types and the existence of exudate and hemorrhage were determined. Also, we addressed the regional distribution of MNV3 lesions between the superior-inferior and the nasal-temporal halves of the macula, and the range of the distance of the lesions from the central fovea. Furthermore, we explored the number of feeding vessels including the cilioretinal artery.

**Results:**

We found 51 lesions in 22 eyes of 21 patients. They were bifocal in 16 (73%) eyes, trifocal in 5 (23%), and quadrifocal in one (4%). No lesion of MNV1 or 2 was found. Fifteen (68%), 2 (9%), and 16 (73%) eyes were associated with retinal hard exudate, subretinal pigment epithelium exudate, and intraretinal hemorrhage, respectively. Thirty (59%) lesions were located in the temporal half of the macula, whereas 21 (41%) were located nasally (*p* = 0.07). One (2%) lesion was closer than 500 µm, 49 (96%) between 500 and 1500 µm, and one (2%) between 1500 and 3000 µm. The lesions were supplied by one arteriole in one (4%) eye, two arterioles in 16 (73%) eyes, and 3 arterioles in 5 (23%) eyes. The CRA contributed as a feeding vessel in 5 (23%) eyes.

**Conclusion:**

The multifocal variant of MNV3 has specific morphological and topographical characteristics. Multimodal imaging allows the understanding of the pathomorphological condition in more detail.


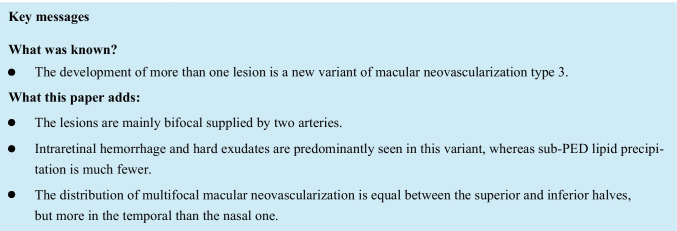


## Introduction

Macular neovascularization type 3 (MNV3), earlier known as retinal angiomatous proliferation, is a type of neovascular age-related macular degeneration (nAMD) with special characteristics. The angiogenic network usually originates from the retinal vessels at first and extends deeply through the retinal pigment epithelium to the subretinal space and anastomose with the underlying choroidal neovessels eventually [1, 2]. Many crucial differences from the other two MNV types have recently been raised, for example, its tendency to distribute in the temporal half of the macula, the contribution of the cilioretinal artery (CRA), and the possible role of disturbance of choroidal blood perfusion and rod damage on its pathogenesis process [3–6]. Moreover, this type affects patients older than those with type 1 or 2 and can be bilateral in up to all cases [7–9].

There is increasing literature about MNV3 including its development and morphological and pathological characteristics in different imaging methods or its response to treatment but these were done on solitary MNV3 [1, 10, 11]. Nonetheless, we found 3 cases of bifocal lesions in our first report on the cilioretinal variant of MNV3 [6]. This outcome led us to initiate an evaluation of multifocal MNV3 (mMNV3) as a variant accounting for 12% of MNV3 cases[12].

In this study, we report on mMNV3. We explore its distribution, morphological and topographical characteristics, and relation to the cilioretinal artery using multimodal imaging.

## Methods

We evaluated consecutive patients with treatment-naïve mMNV3. The patients were participants in different prospective randomized multicenter double blinded studies to evaluate the effectiveness of anti-vascular endothelial growth factor (VEGF) treatment in nAMD in several treatment regimens. Only baseline visits were incorporated in this analysis. Color fundus photography (CFP), optical coherence tomography (OCT), and fluorescein angiography (FA) images were done on all studied eyes. The grading of these studies was carried out at the Vienna Reading Center (VRC). Each image was evaluated by two independent expert readers and in case of disagreement a retina and imaging specialist served as an adjudicator. The diagnosis of mMNV3 was dependent on multimodal imaging methods using FA to make the diagnosis and OCT to confirm it.

### On FA

The presence of leakage emerging from two or more focal hyperfluorescent lesions at an arteriolar-venular shunt [13] or at an abrupt curving of an arteriole into the retinal tissue towards the deeply situated angiogenic network was considered as mMNV3.

### On OCT

**Fig. 1:**
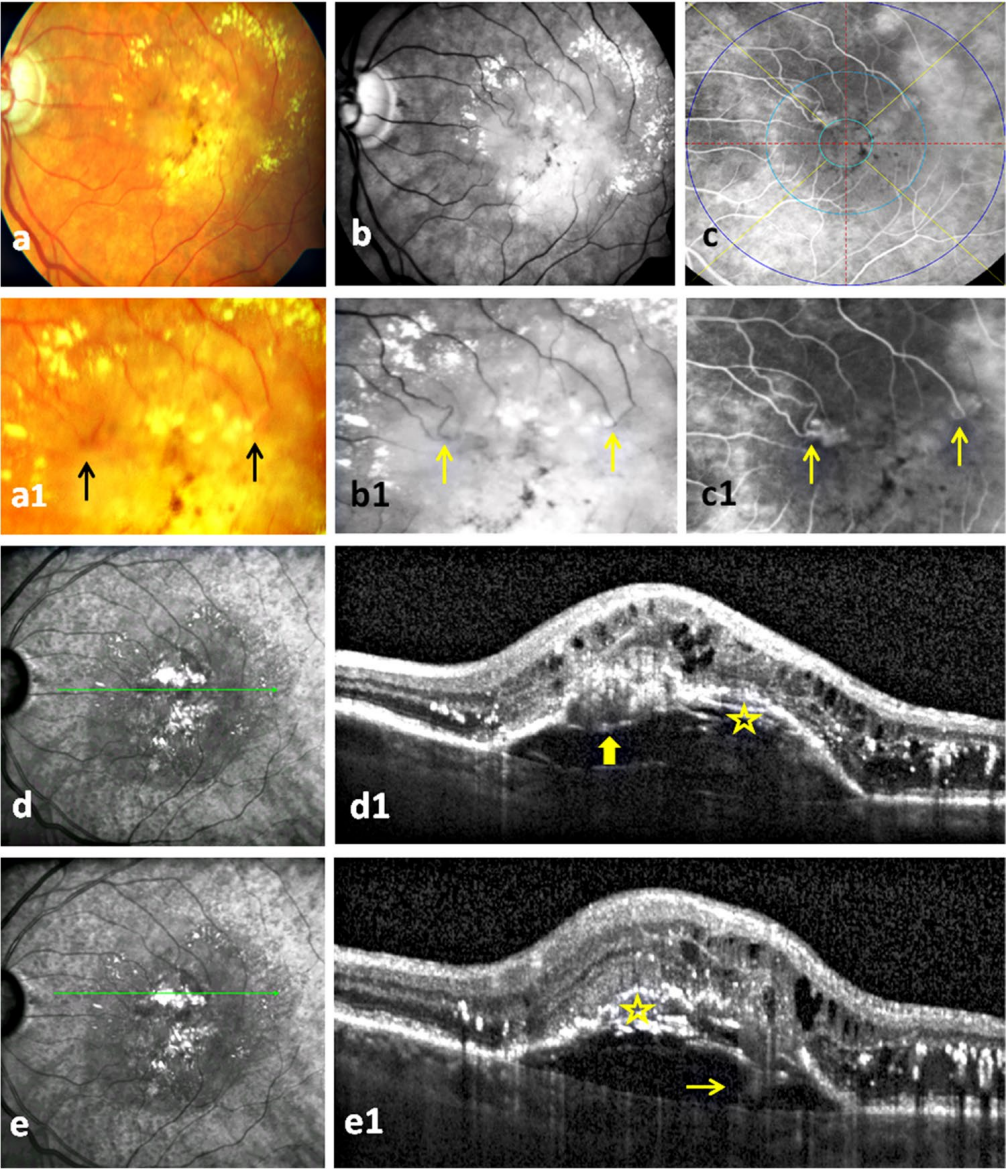
Bifocal macular neovascularization type 3 (MNV3): The first row presents the color fundus photo, red free, and fluorescein angiography (FA) images of massive hard exudates and central pigmentary clumping in a patient with bifocal MNV3 (**a**–**c**). Two MNV3 lesions with focal hyperfluorescence are located in the inner field of ETDRS (i.e., between 500 and 1500 µm from the fovea) in the temporal and nasal parts of the upper half of the macula on an early phase FA (**c**). The second row illustrates bigger images of the first row images which demonstrate the distinguishing retinal arteriolar-venular anastomosis at the nasal lesion and abrupt turning down of an arteriole at the temporal one (2 arrows) (**a1**–**c1**). Of course, FA is superior to other two modalities in identifying these vascular changes (**c1**). The near infrared images and optical coherence tomography scans at (1) the location of the nasal lesion present the characteristic interrupted PED with an overlying hyperreflective mass (thick arrow), intraretinal cysts, and extensive hard exudates (**d**, **d1**). Note the wide spread of pseudodrusen in the outer macula in near infrared images which is very common to find in MNV3 (**d**). (2) At the location of the temporal lesion, they demonstrate the same OCT changes of the nasal lesion and the extension of the hyperreflective band from the retina through the PED to the choroid referring to the retinal-choroidal anastomosis (thin arrow) (**e1**). The onion sign (asterisk) underneath the PED in both scans refers to the extensive lipid deposits (**d1**, **e1**)

**Fig. 2:**
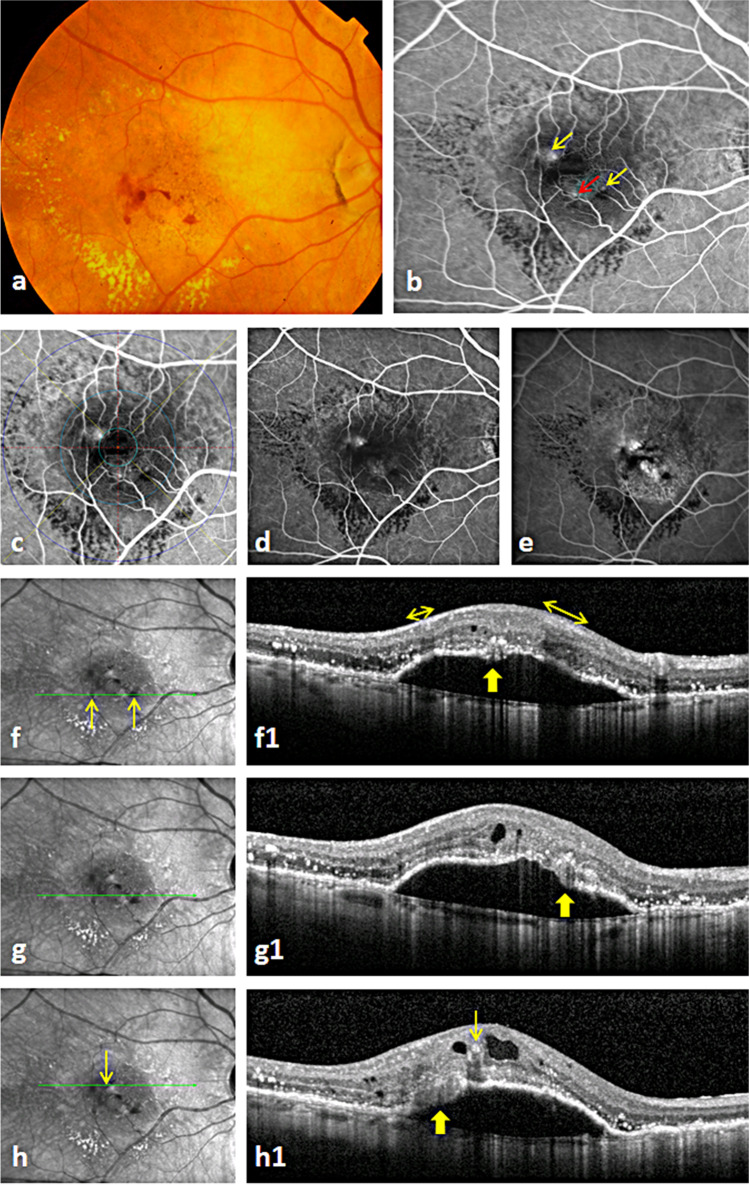
Trifocal macular neovascularization type 3 (MNV3): The color fundus photo shows the characteristic intraretinal hemorrhage and massive hard exudates of MNV3 (**a**). The early phase fluorescein angiography (FA) image highlights the focal hyperfluorescence of three perifoveal MNV3 lesions (red and yellow arrows) fed by three retinal arterioles (**b**). Note that the superficial intraretinal hemorrhages cause coverage of the ends of feeding arterioles of two lesions (2 yellow arrows). The same FA image (**b**) after applying the ETDRS grid showing the distribution of the lesions in the inner field of ETDRS (**c**). The focal hyperfluorescence of the three lesions is easily detectable in mid phase FA (**d**). The late phase FA image presents leakage and a big pigment epithelial detachment (PED) (**e**). Note that the hemorrhage is still well identified due to upper intraretinal position in relation to the underlying leakage (**e**). The infrared images and optical coherence topography scans of (1) the inferior lesion present an interrupted PED with an overlying hyperreflective complex (thick arrow) combined with intraretinal cysts and extensive hard exudates (**f**, **f1**). The intraretinal hemorrhages on near infrared image (2 arrows in **f**) causing thickening of the hyperreflective nerve fiber layer (2 double arrows in **f1**) and shadowing in the underlying layers. (2) The middle lesion with similar retinal changes as in the inferior lesion (**g**, **g1**). (3) The superior lesion with similar OCT changes and characteristic intracystic hemorrhage (thin arrow on **h** and **h1**) and an underlying shadow (**h**, **h1**)

Two or more hyperreflective complexes in the outer retina upon one or more interrupted pigment epithelial detachments (PED) combined with intraretinal cysts [13] in the corresponding OCT scans (Figs. 1 and 2) were considered stage 3 mMNV3 [14]. Presence of lesions at other stages was verified using the classification of MNV3 based on OCT [14].

The number of MNV lesions independently from its type or stage was determined on FA and OCT. The presence of hard exudate imaged as an accumulation of large confluent yellow plaques in the macula was determined on CFP (Figs. 1 and 2). Also, the existence of intraretinal hemorrhage as small splinter-shaped or focal intraretinal hemorrhage on CFP was explored [15]. In case of uncertainty of the exact axial location of a hemorrhage, it was verified as homogenous hyperreflective material which commonly causes underlying shadows on the corresponding OCT (Fig. 2) [15]. We also verified the presence of laminated hyperreflective material (onion sign) as a mark of extensive lipid deposition in the sub-PED space on OCT [16].

The distribution of the lesions in the four quadrants using self-developed custom software which divides the macula in 4 quadrants through vertical and horizontal lines intersecting at the fovea was noted. We compared the distribution of lesions in the macula with expected values given by a uniform distribution using the chi-square test. A 2-sided *p* value lower than 0.05 was considered to refer statistically significant results. Furthermore, the distance of each lesion to the central foveal point was examined using predetermined distances of the three circles of the early treatment diabetic retinopathy study (ETDRS) grid at 500, 1500, and 3000 µm on FA (Figs. 1 and 2). In addition, the number of first-order arterioles feeding the lesions in each eye (Figs. 1 and 2) was determined and the presence of a CRA as a feeding artery was examined on FA.

## Results

We found 22 eyes of 21 patients harboring 51 MNV3 lesions. Bifocal, trifocal, and quadrifocal mMNV3 lesions were seen in 16 (73%), 5 (23%), and one (4%) eyes (Figs. 1 and 2). None of the 22 eyes had type 1 or 2 MNV elsewhere in the macular region on FA images and OCT scans. All lesions were in stage 3 whose OCT showed an interrupted PED with overlying hyperreflective angiogenic complex and extensive intraretinal fluid (Figs. 1 and 2).

### Hard exudate and intraretinal hemorrhage

Fifteen (68%) eyes were associated with hard exudate. Sixteen (73%) eyes showed intraretinal hemorrhage on CFP and OCT (Figs. 1 and 2). No subretinal hemorrhage was noticed.

### Lipid precipitation in the sub-PED space

We identified the onion sign in 2 eyes (9%) of 2 patients on OCT (Fig. 1).

### Topographical distribution of the lesions

Thirty (59%) lesions were located in the temporal half of the macula and 21 (41%) were located in the nasal one (*p* = 0.07). Twenty-five (49%) and 26 (51%) lesions were seen in the superior and inferior halves, respectively (*p* = 0.84). Forty-nine (96%) lesions were found between 500 and 1500 µm distance from central foveal point (Figs. 1 and 2), one (2%) lesion was located between the CFP and 500 µm, and one (2%) between 1500 and 3000 µm.

Considering eyes (and not lesions) by (1) dividing the macula into temporal-nasal halves: 11 (50%) eyes showed their lesions in both the temporal and the nasal halves (Figs. 1 and 2), 8 (36%) had lesions in the temporal half only, and 3 (14%) eyes showed lesions in the nasal half only; (2) dividing the macula into superior-inferior halves: fourteen (64%) eyes showed lesions in both the superior and the inferior halves (Fig. 2), 4 (18%) eyes had their lesions in the superior half only (Fig. 1), and 4 (18%) eyes in the inferior half only.

### Feeding arterioles of the lesions and CRA

mMNV3 lesions were supplied by one (5%) arteriole in one eye, two arterioles (72%) in 16 eyes (Fig. 1), and 3 (23%) arterioles in 5 eyes (Fig. 2). A CRA was found in 8 (36%) of 22 eyes, where it contributed to the blood supply with other retinal arterioles to MNV3 lesions in 5 (23%) eyes only. In the rest 3 (13%) eyes, it was not responsible to provide any blood supply to lesions.

## Discussion

In this study, we evaluated 51 lesions in 22 eyes of 21 patients with mMNV3. The distribution of lesions, prevalence of hard exudates, and intraretinal hemorrhage were addressed. In addition, we explored the possibility of development of other MNV types and the cilioretinal artery of being feeding vessel to the lesions.

Most eyes were associated with hard exudate and intraretinal hemorrhage. We noticed similarly high percentages of exudative changes on both solitary and cilioretinal MNV3 in a previous work [6]. A possible explanation for this morphology could be the higher permeability of the retinal component of the neovascular complex in MNV3 compared with the choroidal NV in MNV1 or 2 [13], and also the intraretinal location of the lesions, where the extravasation from the angiogenic network is not blocked by the outer blood-retinal barrier as it is the case with the choroidal NV. Another possible explanation could be an elevated concentration of lipids and fatty acids in the blood of patients as arterial hypertension is a prominent risk factor in MNV3 compared to other types of MNV [8]. Moreover, the greater incidence of lipid accumulation in the retina (hard exudates) compared to the choroid (onion sign in the sub-PED space) could indicate that the retinal angiogenic component of MNV3 is more responsible of the leakage than the choroidal one. An explanation for the high percentage of intraretinal hemorrhage could be its natural course from the angiogenic network which grows vertically deep into the retina before it extends horizontally within the subretinal space. Therefore, such fragile vertical neovessels are less capable to stretch and tend to bleed as a result from the inevitable swelling of the intraretinal retina[15]. The high presence of intraretinal cysts in all eyes with MNV3 compared to those with other types supports this explanation of the mechanism [13].

Recently, the subretinal pigment epithelial and sub-PED multilaminar hyperreflectivity (onion sign) were found in 12% of eyes with MNV3 and considered to be a marker of growth of more than one MNV3 lesion and poor visual outcome [17]. The authors found that the multifocal lesions were confined to eyes with these changes. In contrast, in our study mMNV3 predominantly existed in eyes without onion sign (Fig. 2).

Patients with MNV3 are usually older than those with other types [2, 10]. Therefore, it was surprising, as in our previous two reports on solitary and cilioretinal MNV3 [3, 6], not to find concurrent MNV1 or 2 lesions elsewhere in the macula, given that all three types share many risk factors and have similar morphological characteristics in early and intermediate AMD. Furthermore, in MNV3, which was first described more than 28 years ago [18], there is still no report to the best of our knowledge about a concurrent development of MNV1 or 2 lesion with MNV3. Therefore, our cumulative data, showing that when one lesion, in multifocal MNV, is MNV3, all other lesions are also MNV3. This finding raises the important question, whether MNV3 has certain genetic or environmental risk factors, which underregulate the development of other MNV types in the same eye [8, 19].

Anti-VEGF in MNV3 is usually found in higher concentration than in the other two types [20]. However, we found that the multifocal variant can demonstrate up to 4 lesions in the same eye. Subsequently, we hypothesize that the development of multiple lesions at the same time might indicate that the stimulus of angiogenesis is more pronounced than it is in case of solitary MNV3.

There is a strong association of occurrence of pseudodrusen with reduction in the choroidal thickness and compromised choroidal perfusion [21–23]. Many studies showed that these features are found in MNV3, in particular in its multifocal variant, more than the other two MNV types [12, 24–26]. Subsequently, we suggest that the reduction in the choroidal flow in case of development of more than one lesion is not localized but there is a generalized ischemic/inflammatory process that ends up with profound production of angiogenic factors [20]. The development of stage 3 lesions in all cases may indicate that the lesions developed simultaneously and support our suggestion of an acute irreversible generalized event.

The distribution of lesions in the temporal half was significantly higher in our first report on 78 solitary MNV3 eyes [3]. Otherwise, the difference in the multifocal variant was insignificant (*p* = 0.07). The insignificant but very low *p* value (0.07) could be due to the relatively small number of cases. Therefore, mMNV3 has also distinct characteristics compared to MNV1 and 2, whose choroidal NV is located subfoveally in about 80% of cases [7]. A recent report evaluated 39 lesions in 18 eyes with mMNV3 [12]. Nonetheless, the authors plotted them with other 130 solitary MNV3 lesions so the individual distribution of multifocal lesion was not explored.

In a recent study using a new three-dimensional OCTA device, the authors found that this novel technique is superior to the standard two-dimensional OCTA and indocyanine green angiography in identifying the “intraretinal angiogenic mass” in patients with MNV3 [27]. The study revealed that each MNV3 lesion represents a complex of one or more intraretinal neovascular tufts emerging from the deep vascular plexus. However, in our study, using OCT and FA only, it was not possible to verify these fine vascular details. Therefore, we considered every intraretinal neovascular complex on OCT or FA as one MNV3 lesion.

CRA is usually one artery which runs from the upper or lower half of the macula towards the corresponding perifoveal area [28]. Therefore, it was not unexpected to find its contribution in the formation of MNV3 as most eyes had bifocal lesions distributed equally in the superior and inferior halves in our study. Its contribution to the blood supply of multifocal lesions was found in quarter of eyes which is within the normal range of existence of CRA in healthy eyes [29, 30]. We noticed a similar finding in our evaluation of 12 MNV3 eyes with CRA feeding vessels [6]. In other words, this supports our results in the first report that CRA does not play a protective factor against development of MNV as it was previously proposed [31]. Instead, we presume that emergence of MNV3 is dependent on the location of a damaged outer retinal area which starts the profound secretion of angiogenic factors rather than which artery (retinal or cilioretinal) spreads over the lesion [20].

The main limitation of our study is the lack of demographic characteristics of the patients such as age and gender as all images were pseudonymized according to the study and grading protocols of the clinical studies evaluated at the VRC. In addition, indocyanine green angiography was not available for the analysis which could have helped to investigate the state of the choroidal blood flow underneath the lesions. Otherwise, our inclusion criteria using specific pathognomonic changes on FA and OCT have also high accuracy in setting the correct diagnosis of MNV3 [13, 14].

In conclusion, in this study we report on a variant of MNV3 describing its main morphological features. mMNV3 tend to be bifocal, distributed equally between the upper and lower half of the macula, and accompanied by hard exudate and intraretinal hemorrhage. Further investigations are needed to explore their response to anti-VEGF and whether they need a higher dosage or a shorter treatment interval.
